# The international space station packed bed reactor experiment: capillary effects in gas-liquid two-phase flows

**DOI:** 10.1038/s41526-023-00302-2

**Published:** 2023-07-18

**Authors:** Mahsa Taghavi, Brian J. Motil, Henry Nahra, Vemuri Balakotaiah

**Affiliations:** 1 Department of Chemical and Biomolecular Engineering, University of Houston, Houston, TX USA; 2 NASA Glenn Research Center, Cleveland, OH USA

**Keywords:** Chemical engineering, Mechanical engineering

## Abstract

Experimental data on flow patterns and pressure drop in two-phase gas-liquid flows through a packed bed obtained aboard the International Space Station (ISS) are analyzed in the limit of low flow rates. Four distinct flow regimes (dispersed bubble, pulse, elongated or large bubble, and gas continuous) are observed and the transition boundaries are identified by a change in the slope of the pressure gradient versus flow rate. It is found that the pressure drop is a function of flow history with the relative magnitude of the hysteresis decreasing with increasing gas or liquid flow rates. Pressure drop (or friction factor) correlations are presented for each of the flow regimes. The capillary or interfacial contribution to the pressure gradient is found to be dominant in the gas channeling regime but comparable to the viscous contribution in the large bubble regime. Preliminary data indicating the slow accumulation of the gas in the bed in the large bubble regime over a longer time period and the intermittent nature of this regime are also presented.

## Introduction

Packed Bed Reactors (PBR) are widely used to carry out many reaction and separation processes in industry because of their low power consumption and compact size compared to other reactor configurations. The typical operation of a packed bed consists of one or more fluids flowing through a fixed bed of solid particles contained within a tube or channel. The solid particles or packing is typically a host for biological growth or a catalytic material where the fluids interact with the packing across the length of the reactor. The packing particle size and shape vary depending on the application to distribute the fluids to flow throughout the interstitial space. The arrangement serves to sustain the chemical or biological reaction taking place within the reactor and minimize operational requirements such as pressure drop. In some cases, it is required to minimize the localized shear on the solid packing. Due to its relative size, versatility, reliability, and low operational power, a packed bed is a viable unit operation in support of deep-space missions. Importantly, most reactor beds for space applications operate using very low liquid flow rates^1^ where the operating parameters such as pressure gradient, and phase distribution are dominated by capillary and viscous forces. Some examples in which a PBR is used for space applications include the Volatile Removal Assembly (VRA)^1,2^, the Integrated Advanced Water Recovery System (AWRS)^3,4^, and the IntraVenous Water GENeration system (IVGEN)^5,6^.

NASA funded a series of experiments to develop a fundamental understanding of how PBRs perform in the microgravity environment. Two separate test series were initially conducted on a reduced gravity aircraft test platform which limits the low gravity test duration to <20 s^7^ followed by two additional test series, named the Packed Bed Reactor Experiment (PBRE), on the International Space Station (ISS)^8,9^ which allowed for much longer duration testing at very low (micro) gravity conditions.

In the aircraft experiments, by changing the particle diameter, liquid viscosity, and both gas and liquid flow rates, a form of the modified Ergun equation^10^ was developed for predicting pressure drop in microgravity conditions at moderate to high gas and liquid flow rates where inertial forces played a more significant role. A flow regime map and a criterion for predicting dispersed bubble to pulse flow transition were also developed. However, the limited duration of microgravity time (<20 s) in these experiments did not provide enough time to fully develop steady state conditions at the lower flow rates typical for space-based reactors. This led to the development of the PBRE for testing on the ISS to obtain experimental results in the range of flow rates of interest. These systems operate at flow rates that require several minutes to an hour to reach steady flow. Two separate ISS flights were conducted for the PBRE and the design and test conditions are discussed in section RESULTS.

In the first series of the PBRE experiments^8^, the bed was initially flushed with liquid only before each new flow condition to establish similar initial conditions. An extended modified Ergun equation was developed for predicting the pressure drop in the viscous-capillary (V–C) regime. Moreover, a bubble to pulse flow regime map was obtained based on the experimental data which also reasonably verified the aircraft flow transition correlation. In the developed modified Ergun equation, the overall pressure gradient was the summation of viscous, inertial, and capillary or interfacial contributions. The capillary contribution of the pressure drop for the wetting glass particles was compared with the non-wetting Teflon particles in the V–C regime. It was found that the capillary contribution was dominant for the wetting particles whereas the viscous contribution was dominant for the non-wetting particles.

The second series of the PBRE experiments (PBRE-2)^9^ was essentially a reflight of the original PBRE except for a smaller packing size and several modifications to improve the accuracy and control of the flow loop. Moreover, both gas flush and liquid flush pre-flows were used before the tests. Higher gas hold-up and pressure gradients were observed for the liquid flush compared to the gas flush tests. A comparison between the operational parameters and fluid properties used in PBRE and PBRE-2 experiments is presented in Supplementary Table 1. Using pressure data obtained for PBRE-2, a modified correlation was developed for predicting the two-phase pressure gradient in packed beds under the microgravity condition operating outside of the V–C regime. It was found that the capillary forces were the dominant contributor to pressure drop throughout the tested flow rate range and was linear with superficial liquid velocity but was much weaker function of superficial gas velocity. It was also a function of the particle size and varied inversely with the particle diameter^9^. The pressure data at the lower gas-liquid flow rates (V–C regime) measured during the PBRE-2 series of experiments and the same at higher flow rates for PBRE experiments were not analyzed in earlier works. In the current work, we review all the available microgravity PBRE data, analyze the V–C pressure gradient data, classify the flow regime map more accurately, and propose a two-phase friction factor/ pressure gradient correlation for each flow regime. Moreover, we present here the longer duration pressure drops measurement data as well as the hysteresis experimental data.

## Results

### Experiment and test conditions

**Fig. 1:**
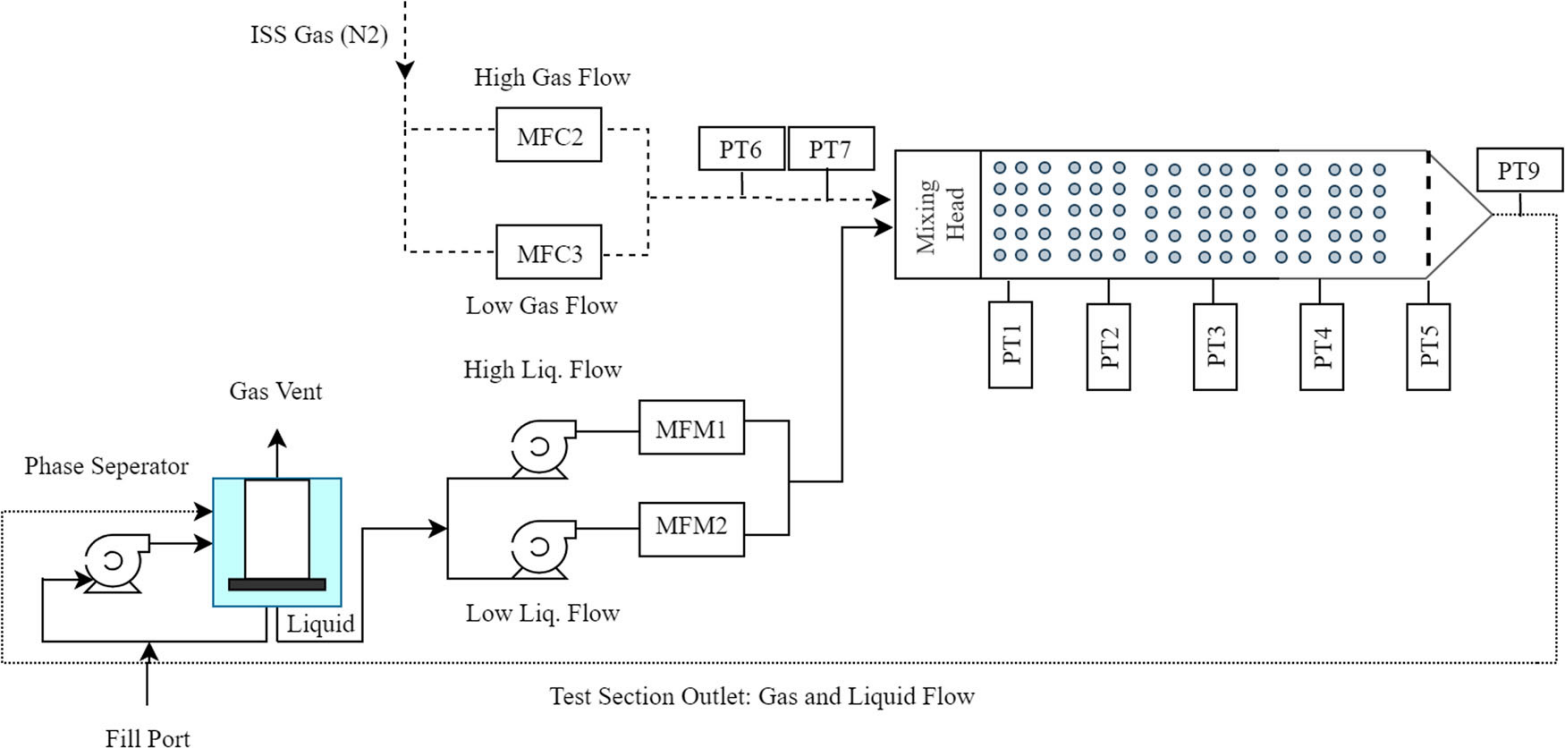
Schematic diagram of the flow loop in the Packed Bed Reactor Experiment.

Two different test columns were flown during the 0-g aircraft testing using the same flow loop and similar diagnostics which are described in detail in Motil et al.^7^. The first ISS test series, named Packed Bed Reactor Experiment (PBRE), included two identical test columns: one packed with 3 mm spherical glass beads and the other packed with 3 mm Teflon beads; Details are presented in Motil et al.^8^. The packing materials for these initial tests were selected to compare flow patterns and pressure drop between a wetting material (glass) and a non-wetting material (Teflon). A detailed description of the experiments and the results of the PBRE are discussed in Motil et al.^8^. Over 495 steady-state test points were obtained for the glass packing and 187 steady-state test points for the Teflon packing. The experiments were conducted in the Microgravity Science Glovebox facility with 5.08 cm diameter cylindrical test sections that were 60 cm long. The test columns were constructed out of clear polycarbonate material for flow visualization and the randomly distributed packing was held in place using spring-loaded perforated end caps. The basic flow loop (Fig. 1) provided nitrogen gas which was mixed with pure water and separated downstream of the test section and vented to the cabin. Multiple gas and liquid flow loops were required to accurately span the full range of flow conditions. Five absolute pressure transducers were evenly spaced along the column and two high-speed video cameras recorded the entrance region and a fully developed region in the middle section of the column. The average of the pressure data collected in a specified time interval was calculated for each pressure transducer and the pressure difference between the first and fifth transducer was reported as the bed pressure drop.

The second ISS test series was named PBRE-2 and is discussed in detail in Taghavi et al.^9^. PBRE-2 uses the same column with smaller (2 mm diameter) glass beads. The smaller beads were still within the range typical of reactors, but the intent was to increase the pressure drop along the column to enable more accurate pressure readings at lower flow rates. Other modifications before the PBRE-2 reflights included the removal of several check valves and a shortening of the mixing section upstream of the columns – both of which were thought to be contributing to pressure oscillations in the flow loop at the higher liquid flow rates. These modifications solved the external pressure fluctuations and we were able to obtain steady inlet conditions at much higher liquid flow rates.

In all PBRE-2 experiments, a pre-flow “liquid flush” was used to establish similar initial conditions before testing. As the procedure is explained in the earlier publication^9^, it consists of flowing liquid at 150 kg h^−1^ for 30 s, followed by 20 kg h^−1^ for 120 s, and then flowing the selected gas and liquid flow rates dictated by test conditions for a duration long enough to establish pseudo-steady flow conditions throughout the column. For each run, and after the liquid or gas flush period is completed, the selected liquid and gas test flows are applied and controlled until an equivalent of 150% of the bed void volume was passed through the column to ensure pseudo-steady flow conditions were achieved prior to collecting the pressure data.

The second bed flown with PBRE-2 included 3–3.5 mm Alumina packing to apply the results presented here to a realistic packing material. Detailed analysis of the Alumina bed is still underway and is only mentioned briefly here to support the main conclusion. In addition, a known challenge with using Alumina packing is the buildup of Alumina fines within the water loop. The PBRE loop was not designed to remove the fines, so we limited the pre-flow conditions. Even with this limitation, after a short period of testing, the low-flow water loop became plugged with Alumina which did not allow us to complete the full test matrix.

### Flow regime map in microgravity

**Fig. 2:**
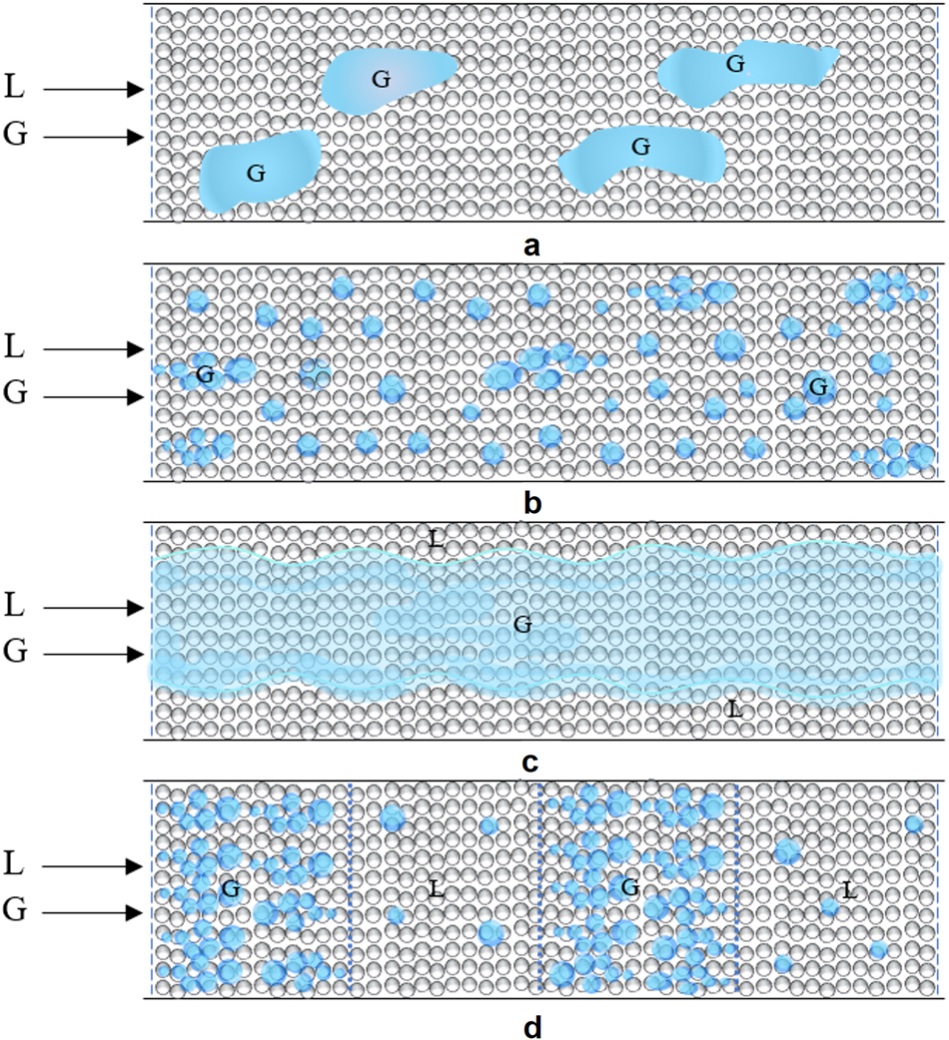
Schematic diagrams of the flow regimes. **a** Elongated/large bubble; **b** Dispersed bubble; **c** Gas channeling; **d** Pulse regime.

Most of the flow maps presented in the literature for the normal gravity gas-liquid downward flow in packed beds are based typically on visual observations or subjective methods^11–14^. There are a few objective methods to predict the transition from a bubbly to a pulsing pattern based on the time series analysis of pressure fluctuations at the bottom of the column^15–17^. These methods are based on indicators such as a sudden increase in the intensity of a frequency component in the power spectrum; a sudden increase in the standard deviation of the pressure fluctuations; and auto-correlation and cross-correlation functions of the pressure signal. For columns operated in microgravity, Motil et al.^7^ used a method in their aircraft experiments based on the sudden increase in the intensity of frequency components in the power spectrum of the pressure fluctuations. Later in their PBRE experiments, they used a combined visual and power spectrum-based analysis for defining the transition boundary between the bubble and pulse flow patterns and found a reasonable consistency of the PBRE experimental data with the aircraft experiment flow transition correlation^8^. In the PBRE-2 experiments and in analyzing the more accurate pressure traces and flow regime video recordings, Taghavi et al.^9^ detected two other flow regimes in addition to the dispersed bubble and pulse flow regimes, which occurred at low liquid flow rates and were called large or elongated bubble and gas channeling flow regimes. Schematics of these flow regimes are depicted in Fig. 2.

**Fig. 3:**
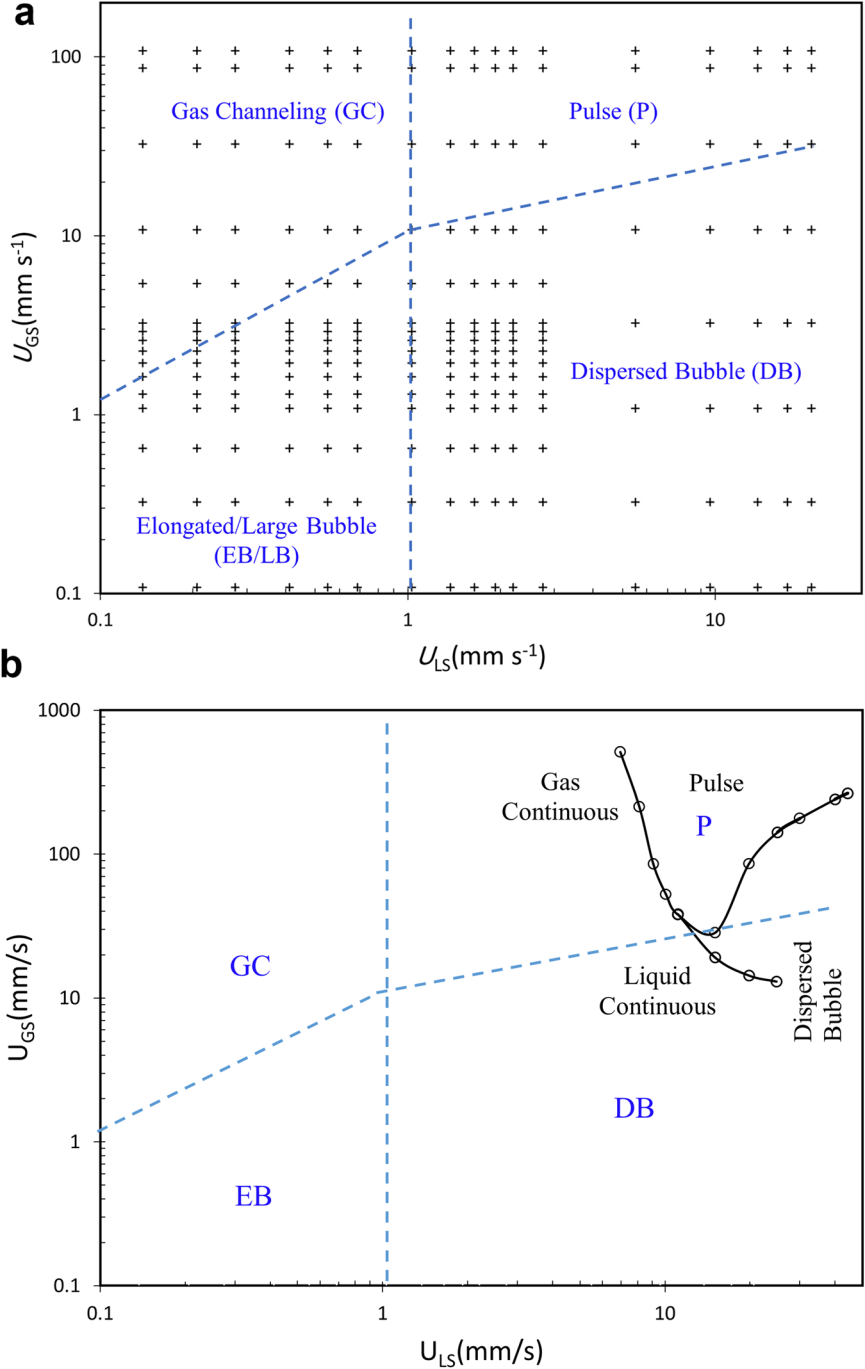
Flow regimes map. **a** Approximate map for the water-N2 system observed in the microgravity PBRE experiment with 2 mm packing size [Plus signs represent the experimental test matrix]; **b** Comparison of the microgravity flow map (dashed lines) with the normal gravity cocurrent downflow map (solid lines).

In the current work, we propose an objective method based on a change in the slope of the pressure gradient (or capillary contribution to the pressure gradient) when it is plotted versus the gas or liquid flow rate or Reynolds numbers. Using this method, we updated our earlier microgravity flow map^9^ and identified more accurate flow regime boundaries (Fig. 3a and Supplementary Fig. 1).

**Fig. 4:**
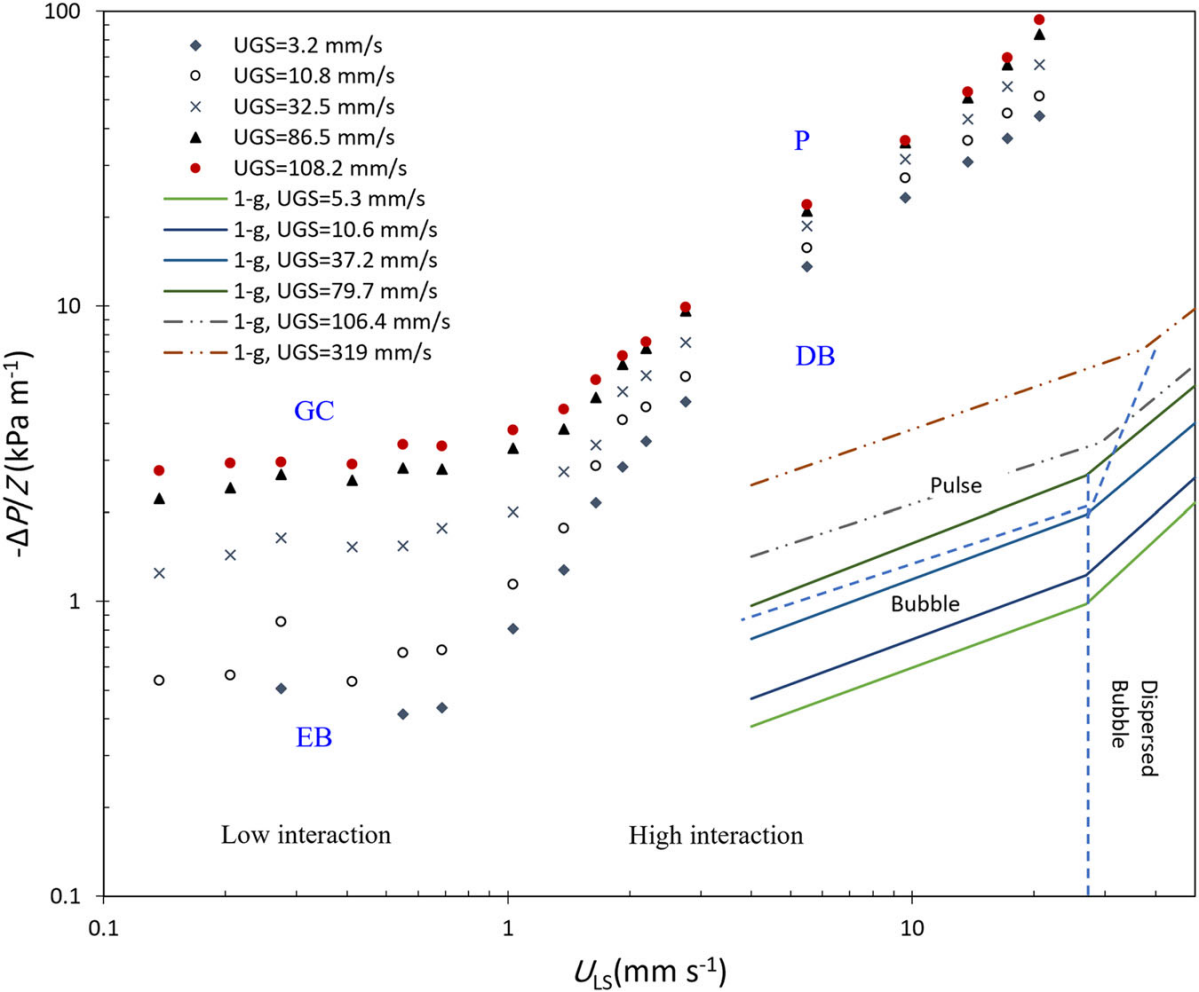
Measured pressure gradient versus superficial liquid velocity at microgravity and comparison with the normal gravity data.

**Fig. 5:**
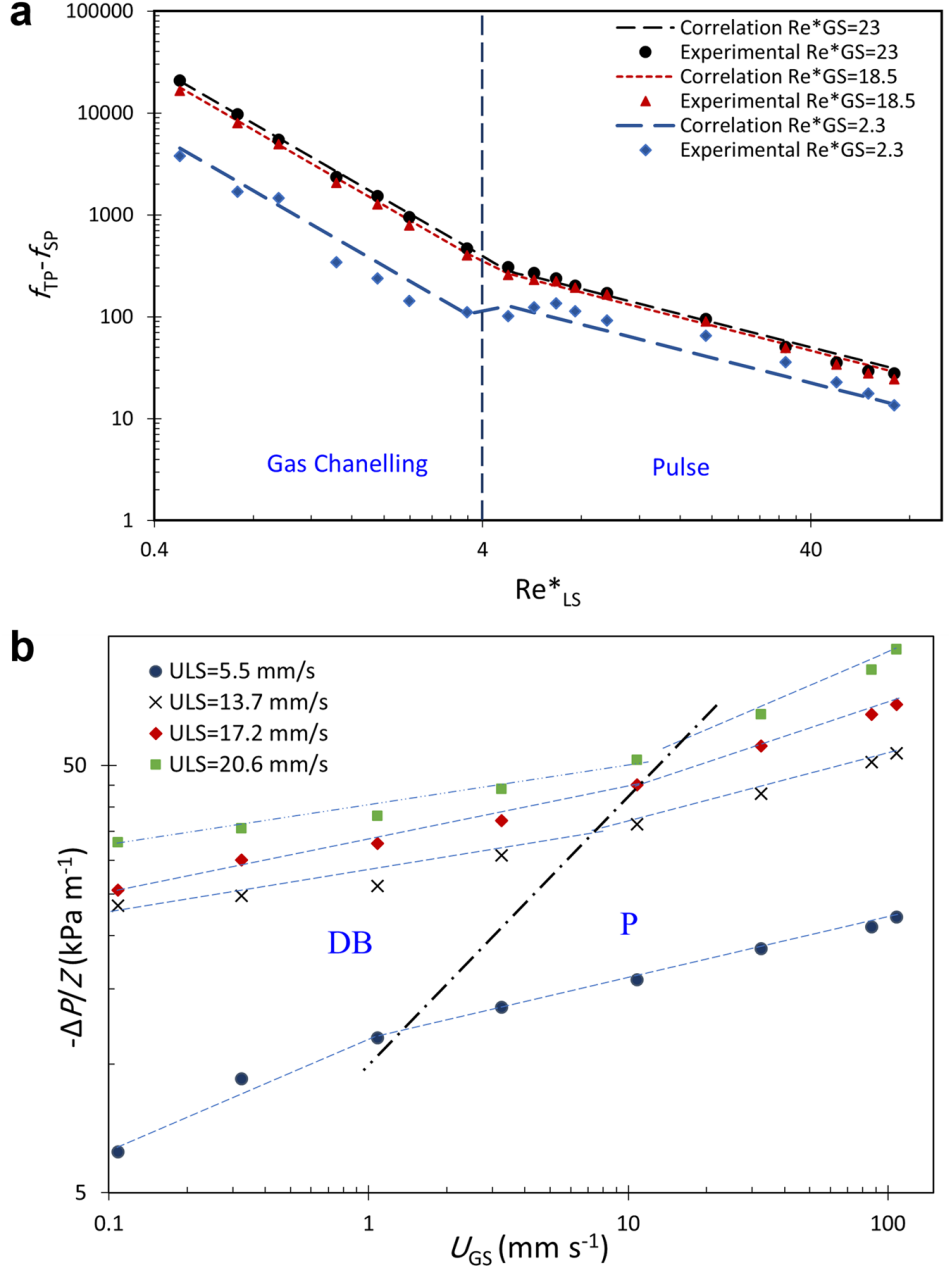
Flow regime transition with slope change. **a** Measured and calculated capillary friction factor versus modified liquid Reynolds number at three fixed modified gas Reynolds numbers; **b** Measured pressure gradient versus gas superficial velocity.

It is observed that the transition from the low interaction regime (large bubble and gas channeling) to the high interaction regime (dispersed bubble and pulse) occurs at about *U*_LS_ ≈ 1 mm s^−1^ or Re^*^_LS_ = 3.6 independent of the gas superficial velocity using the slope change criterion shown in Figs. 4 and 5a. The transition from the dispersed bubble to the pulse regime occurs within the high interaction region by increasing the gas flow rate. The approximate transition boundary was determined using the slope change shown in Fig. 5b. Visual observation of captured videos from the test matrix also verified that the four flow regimes are located inside the defined boundaries. The same method was used for approximating the boundary between the elongated or large bubble regime to the gas channeling regime occurring within the low interaction region.

### Comparison with the normal gravity cocurrent downflow map

In Fig. 3b we have compared our microgravity flow map with the normal gravity one presented by Tosun^11^. Tosun also observed four different flow regimes in his 1-g cocurrent downflow experiments with 1.9 mm size glass beads in an air-water system. In his flow map, the gas continuous regime is equivalent to our 0-g gas channeling regime, and his liquid continuous regime is equivalent to our elongated or large bubble regime. As it is shown in Fig. 3b, there is a good overlap between the two data series in the pulse and dispersed bubble flow regimes at high liquid flow rates. However, the normal gravity data for the cocurrent downflows are limited to the high gas and liquid flow ranges and practically it is not possible to have a cocurrent gas–liquid downflow at such low flow rates as those used in the microgravity experiments. For this purpose, the minimum liquid velocity should be higher than the terminal velocity of a rising bubble in a porous media which was reported to be ~167–202 mm s^−1^ by Roosevelt and Corapcioglu^18^ for bubble sizes larger than 2 mm in a porous medium filled with 4 mm glass beads. Corapcioglu et al.^19^ also developed a theoretical model for predicting bubble rise velocity in a porous media. Using their model, the terminal velocity of a 2 mm bubble in a 2 mm glass beads media is calculated to be around 70 mm s^−1^. Therefore, the minimum liquid velocity should be higher than 70 mm s^−1^ in such a bed for having a cocurrent downflow. However, in our microgravity experiments due to the lack of gravity forces, we could establish liquid superficial velocities as low as 0.1 mm s^−1^ and observe the formation of large bubbles encapsulating several particles. For the cocurrent upflow case, Taghavi and Balakotaiah^20^ performed experiments in a liquid-filled bed of various particle sizes at the limit of low gas flow rates and reported the formation of large or elongated bubbles having a maximum diameter of 12 times the packing diameter at low Bond numbers, the same as the elongated bubbles observed at the low liquid and gas flow rates (EB regime) in the microgravity experiments. Murugesan and Sivakumar^21^ also performed gas-liquid cocurrent upflow experiments using 15.7 mm spherical particles and reported having three flow regimes of bubble, pulse, and dispersed bubble. Their pulse flow regime seems not to be accurate enough since they have not distinguished this regime from a gas channeling regime which occurs at lower liquid flow rates than the pulse regime. Further comparison between flow regime maps with 1-g up and downflow and 0-g is presented in Supplementary Fig. 2.

Because of the normal gravity experimental limitations, the developed flow map in the microgravity environment is unique and is the only flow map reporting the flow regime at very low gas and liquid flow rates and covers a wider flow range from low to high flow rates.

### Single-phase flow in porous media in microgravity

To determine the single-phase Ergun equation coefficients (Eq. 1), liquid-only flow experiments were conducted by flooding the column with liquid.

1 $$\frac{-\Delta P}{Z}={C}_{\text{V}}\frac{{(1-\varepsilon )}^{2}}{{\varepsilon }^{3}}\frac{{\mu }_{\text{L}}{U}_{\text{LS}}}{{d}_{\text{p}}^{2}}+{C}_{\text{I}}\frac{\left(1-\varepsilon \right)}{{\varepsilon }^{3}}\frac{{\rho }_{\text{L}}{U}_{\text{LS}}^{2}}{{d}_{\text{p}}} , {f}_{\text{SP}}=\frac{{C}_{\text{V}}}{{\text{Re}}_{\text{LS}}^{* }}+{C}_{\text{I}}$$

The constants *C*_V_, *C*_I_, and *ε* in the pressure gradient equation were estimated as *C*_V_ = 150.8, *C*_I_ = 1.78, *ε* = 0.358, as reported by Taghavi et al.^9^.

### Two-phase flow in porous media in microgravity

Many empirical pressure drop models still in use today use a variation of the approach developed by Lockhart and Martinelli^22^ (L–M) for gas-liquid flow in open channels (without packing). In this approach, the total pressure drop is formulated by taking the two-phase pressure drop due to friction as the pressure drop that would arise from either phase flowing alone, multiplied by some factor *Φ*^*2*^_L_ and *Φ*^*2*^_G_. *Φ*^*2*^ is then plotted versus the square root of the ratio of the liquid pressure drop to the gas pressure drop, *χ* to develop the best fit [*χ* is known as the Martinelli parameter].

**Fig. 6:**
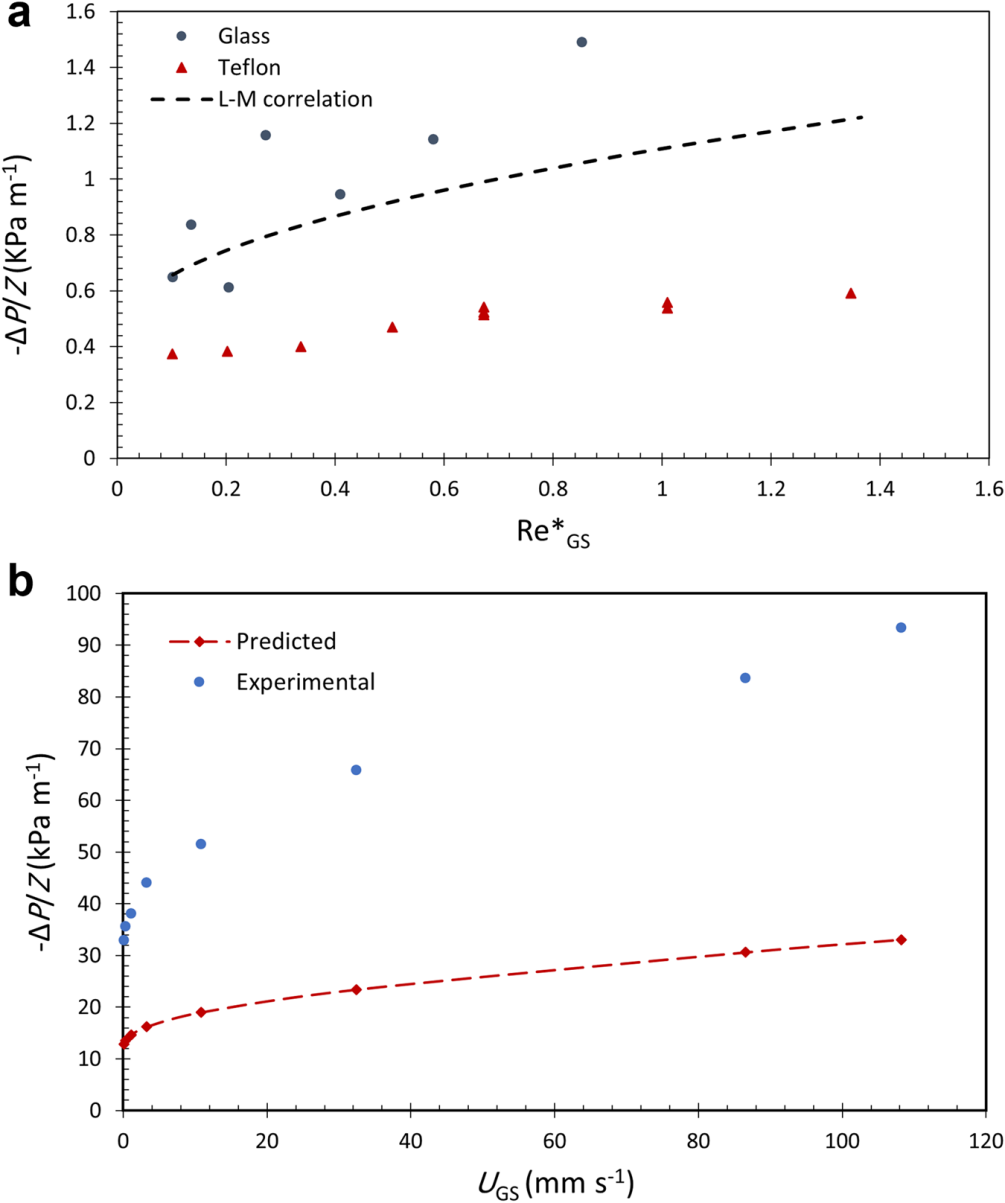
Comparison of the experimental PBRE/PBRE2 pressure gradient data for the glass and Teflon particles. **a** Lockhart–Martinelli approach; **b** Macro-scale momentum balance approach.

A well-known example is presented by Larkins et al.^23^ using Raschig rings and spheres. Unfortunately, this approach requires capillary forces to be neglected. Charpentier and Favier^24^ addressed capillary liquid holdup by correlating it to the Eötvos (or Bond) number. These results have been refined over the years such as the work of Tosun^11,12^, but are generally restricted to the trickle flow regime only. Pinna et al.^25^ provide a good summary of pressure drop models and the theoretical basis for applying the L–M approach. In the microgravity environment, capillary forces dominate in nearly all flow regimes which makes this approach ineffective. A comparison between the experimental data obtained in PBRE experiments for both glass and Teflon packings with the L–M approach for the gas–liquid pressure gradient is presented in Fig. 6a. For the L–M correlation, we used the coefficients fitted by Tosun^12^:

2 $${\varPhi }_{\text{L}}=1+\frac{1}{\chi }+\frac{1\text{.}424}{{\chi }^{0\text{.}576}},$$

It is observed that this approach cannot predict well the microgravity pressure gradient, especially the dependence on the gas Reynolds number.

The second class of models used in the literature to predict two-phase pressure drop use macro-scale momentum balances for each phase along with relative permeability (for viscous term) and passability (for inertia term), and interfacial drag^26–28^. In this approach, the pressure gradient in each phase is expressed as

3 $$-{\left(\frac{\partial P}{\partial z}\right)}_{\text{L}}=\frac{{\mu }_{\text{L}}}{\kappa {\kappa }_{\text{L}}}{U}_{\text{LS}}+\frac{{\rho }_{\text{L}}}{\eta {\eta }_{\text{L}}}{U}_{\text{LS}}^{2}+\frac{{F}_{\text{i}}}{1-\alpha },$$

4 $$-{\left(\frac{\partial P}{\partial z}\right)}_{\text{G}}=\frac{{\mu }_{\text{G}}}{\kappa {\kappa }_{\text{G}}}{U}_{\text{GS}}+\frac{{\rho }_{\text{G}}}{\eta {\eta }_{\text{G}}}{U}_{\text{GS}}^{2}+\frac{{F}_{\text{i}}}{\alpha },$$

where the bed permeability ($\kappa$) and passability ($\eta$) are given by

5 $$\kappa =\frac{{\varepsilon }^{3}{d}_{{\rm{p}}}^{2}}{150.8{\left(1-\varepsilon \right)}^{2}},\eta =\frac{{\varepsilon }^{3}{d}_{p}}{1.78\left(1-\varepsilon \right)}.$$

Here, $\alpha$ is the gas holdup, and the relative permeabilities of gas and liquid (${\kappa }_{{\rm{G}}}$, ${\kappa }_{{\rm{L}}}$), as well as passabilities (${\eta }_{{\rm{G}}}$, ${\eta }_{{\rm{L}}}$), are empirical functions of $\alpha$, $U$_LS_, and $U$_GS_. The interfacial drag ${F}_{{\rm{i}}}$ is also an empirical function of $\alpha$ and other parameters. The value of $\alpha$ is determined by equaling the pressure gradients predicted by Eqs. 3 and 4. Once $\alpha$ is known, the two-phase pressure gradient can be computed by either Eqs. 3 or 4 and neglecting the capillary pressure.

We note that this approach has never been tested using data under microgravity conditions. Further, for all the empirical models proposed in the literature, the interfacial drag is proportional to the acceleration due to gravity (*g*) and hence is zero under microgravity conditions. Taking ${F}_{i}\,$= 0 and using the empirical relations^27,28^

6 $${\kappa }_{\text{L}}={\left(1-\alpha \right)}^{3},\,{\kappa }_{\text{G}}={\alpha }^{3}$$

7 $${\eta }_{\text{L}}={\left(1-\alpha \right)}^{m},{\eta }_{\text{G}}={\alpha }^{m},(\text{m}=\mathrm{3,5},\text{or}\,6).$$

We have tested the applicability of this model to our data. The results are summarized in Fig. 6b. For example, at the highest gas and liquid flow rates used in our experiments, it is found that the predicted pressure drop is only about 30% of the measured value. Thus, we conclude that this model underestimates the capillary contribution to the pressure drop which can reach ~85% of the total pressure gradient at high flow rates^9^.

In the third approach discussed by Motil et al.^7^, the two-phase pressure drop in porous media is expressed as the sum of an Ergun-type single-phase friction factor and a “dynamic phase interaction term” that accounts for the associated capillary effects i.e.

8 $${{f}}_{\rm{TP}}\,={{f}}_{\rm{SP}}+{{C}_{\rm{S}}\left({\rm{Re}}_{\rm{GS}}^{* }\right)}^{{\alpha }}{({\rm{Re}}_{\rm{LS}}^{* })}^{{\beta }}{\rm{Su}}_{\rm{L}}^{{\gamma }},$$

9 $${f}_{\text{TP}}\,={{f}}_{\text{SP}}+{{C}_{\text{S}}\left({\text{Re}}_{\text{GS}}^{* }\right)}^{{\alpha }}{({\text{Ca}}_{\text{LS}}^{* })}^{{\beta }}{\text{Su}}_{\text{L}}^{\,{\beta }+{\gamma }}$$

10 $${f}_{\text{TP}}\,=\frac{{C}_{\text{V}}+{{C}_{\text{S}}\left({\text{Re}}_{\text{GS}}^{* }\right)}^{{\alpha }}{({\text{Re}}_{\text{LS}}^{* })}^{{\beta }+1}{\text{Su}}_{\text{L}}^{{\gamma }}}{{\text{Re}}_{\text{LS}}^{* }}+{C}_{{\rm{I}}},$$

or in dimensional form

11 $$\begin{array}{l}\frac{-\Delta P}{Z}={C}_{{\rm{V}}}\frac{{(1-\varepsilon )}^{2}}{{\varepsilon }^{3}}\frac{{\mu }_{L}{U}_{{\rm{LS}}}}{{d}_{{\rm{p}}}^{2}}+{C}_{{\rm{I}}}\frac{\left(1-\varepsilon \right)}{{\varepsilon }^{3}}\frac{{\rho }_{{\rm{L}}}{U}_{{\rm{LS}}}^{2}}{{d}_{{\rm{p}}}}\\\qquad\quad\;\,+\,{C}_{{\rm{S}}}\frac{\left(1-\varepsilon \right)}{{\varepsilon }^{3}}\left(\frac{{\rho }_{{\rm{L}}}{U}_{{\rm{LS}}}^{2}}{{d}_{{\rm{p}}}}\right){\left(\frac{{\rho }_{{\rm{G}}}{U}_{{\rm{GS}}}{d}_{{\rm{p}}}}{{\mu }_{{\rm{G}}}(1-\varepsilon )}\right)}^{{\alpha }}{{\left(\frac{{\rho }_{{\rm{L}}}{U}_{{\rm{LS}}}{d}_{{\rm{p}}}}{{\mu }_{{\rm{L}}}(1-\varepsilon )}\right)}^{{\beta }}\left(\frac{{{d}_{{\rm{P}}}\rho }_{{\rm{L}}}\sigma }{{\mu }_{{\rm{L}}}^{2}}\right)}^{\!{\gamma }}\end{array}$$

The dimensionless numbers in the equations above are defined as follows

$${\text{Re}}_{\text{LS}}^{* }=\frac{{\rho }_{\text{L}}{U}_{\text{LS}}{d}_{\text{p}}}{{\mu }_{\text{L}}\left(1-\varepsilon \right)}{\rm{Modified\; liquid\; Reynolds\; number}}$$

$${\text{Re}}_{\text{GS}}^{* }=\frac{{\rho }_{\text{G}}{U}_{\text{GS}}{d}_{\text{p}}}{{\mu }_{\text{G}}\left(1-\varepsilon \right)}{\rm{Modified\; gas\; Reynolds\; number}}$$

$${\text{Su}}_{\text{L}}=\frac{{\text{Re}}_{\text{LS}}^{* }}{{\text{Ca}}_{\text{LS}}^{* }}=\frac{{d}_{\text{p}}{\rho }_{\text{L}}\sigma }{{\mu }_{\text{L}}^{2}}{\rm{Suratman\; number}}$$

$${\text{Ca}}_{\text{LS}}^{* }=\,\frac{{\mu }_{\text{L}}{U}_{\text{LS}}}{\sigma \left(1-\varepsilon \right)}{\rm{Capillary\; number}}$$

The two-phase friction factor is dependent on different variables hidden inside the above dimensionless numbers including gas and liquid superficial velocity, density, viscosity, and surface tension, as well as the particle size and bed porosity. In PBRE experiments, we only varied gas and liquid flow rates and used two particle sizes. Since the liquid Suratman number was not varied by more than a factor of 2 in these experiments, the exponent on the Suratman number in Eqs. 8–11 and all the other developed PBRE equations was taken to be the same as that obtained in the aircraft-based experiments which covered a wide range of Suratman numbers by varying liquid viscosity, surface tension, and particle size. As the pressure gradient due to interfacial effects should increase with both the gas flow rate and the liquid flow rate, the exponents α and β are expected to be positive in Eq. 11.

We note that introducing any gas at a fixed liquid flow rate can impact the observed pressure gradient in two ways: the first is the reduction in the flow area available for the liquid (thus enhancing the viscous and/or inertial contribution to the pressure gradient), and the second is the capillary (or interfacial) contribution. The capillary contribution is due to pressure (or energy) loss caused by gas-liquid interfacial friction and also the passage of gas bubbles through the pores by repeated contraction and expansion. In our view, it is not possible to separate the contributions of different mechanisms, especially in gas-liquid flows. Further, under the conditions of our experiments (low gas and liquid flow rates), the capillary/interfacial contribution is dominant. For lack of better terminology, we refer to the last term of Eq. (11) as the capillary or interfacial contribution to the pressure gradient. [Remark: pressure drop due to capillary effects should not be confused with “capillary pressure” which is the difference in the pressure at the gas-liquid interface].

For the high interaction region which covers pulse and dispersed bubble flow regimes, the exponent on the modified liquid Reynolds number (or the Capillary number) and modified gas Reynolds number in Eqs. 8–11 was estimated as *α* = 0.2, and *β* = −1 using data fitting of PBRE-2 pressure drop data^9^. However, these exponents could not predict the two-phase friction factor accurately when the whole data series were considered. We found out that a single correlation cannot predict the two-phase pressure gradient over all the different flow regimes accurately enough. Therefore, we classified data into four different flow regimes using the modified flow regimes boundaries (Fig. 3a) and developed a specific correlation for predicting the two-phase friction factor and pressure gradient in each flow regime. Using non-linear regression of the complete data set of the low interaction region which covers large bubble and gas channeling regimes, with pressure gradient values beyond the accuracy of the measurements, this correlation was found to fit the data well with *α* = 0.24, *β* = −1.83, *C*_*S*_ = 0.26 for the big bubble regime (Eqs. 12–13), and *α* = 0.66, *β* = −1.86, *C*_*S*_ = 0.21 for the gas channeling regime (Eqs. 14–15). Equations 12 and 14 are based on dimensionless numbers (friction factors and Reynolds numbers), whereas Eqs. 13 and 15 are based on the capillary pressure gradient and superficial velocities. The total pressure gradient is the summation of a single-phase pressure gradient which includes viscous and inertial contributions plus the two-phase capillary contribution. Therefore, the capillary contribution of the pressure gradient (Eqs. 13 and 15) is obtained by subtracting the single-phase (liquid-only) pressure gradient from the total pressure gradient. In the same way, we define the capillary friction factor as the subtraction of the single-phase friction factor from the two-phase friction factor.

12 $${f}_{\text{TP}}-{f}_{\text{SP}}=0\text{.}26\,{({\text{Re}}_{\text{GS}}^{* })}^{0\text{.}24}{({\text{Re}}_{\text{LS}}^{* })}^{\text{-}1\text{.}83}{\text{Su}}_{\text{L}}^{2/3}$$

13 $${\left(\frac{-\Delta P}{Z}\right)}_{\!\text{capillary}}=0\text{.}26\frac{\left(1-\varepsilon \right)}{{\varepsilon }^{3}}\left(\frac{{\rho }_{\text{L}}{U}_{\text{LS}}^{2}}{{d}_{{\rm{P}}}}\right){\left(\frac{{\rho }_{\text{G}}{U}_{\text{GS}}{d}_{\text{p}}}{{\mu }_{\text{G}}(1-\varepsilon )}\right)}^{0\text{.}24}{{\left(\frac{{\rho }_{\text{L}}{U}_{\text{LS}}{d}_{\text{p}}}{{\mu }_{\text{L}}(1-\varepsilon )}\right)}^{-1\text{.}83}\left(\frac{{{d}_{{\rm{p}}}\rho }_{\text{L}}\sigma }{{\mu }_{\text{L}}^{2}}\right)}^{2/3}$$

14 $${f}_{\text{TP}}-{f}_{\text{SP}}=0\text{.}21\,{({\text{Re}}_{\text{GS}}^{* })}^{0\text{.}66}{({\text{Re}}_{\text{LS}}^{* })}^{\text{-}1\text{.}86}{\text{Su}}_{\text{L}}^{2/3}$$

15 $${\left(\frac{-\Delta P}{Z}\right)}_{\!\text{capillary}}=0\text{.}21\frac{\left(1-\varepsilon \right)}{{\varepsilon }^{3}}\left(\frac{{\rho }_{\text{L}}{U}_{\text{LS}}^{2}}{{d}_{\text{p}}}\right){\left(\frac{{\rho }_{\text{G}}{U}_{\text{GS}}{d}_{\text{p}}}{{\mu }_{\text{G}}(1-\varepsilon )}\right)}^{\!0\text{.}66}{{\left(\frac{{\rho }_{\text{L}}{U}_{\text{LS}}{d}_{\text{p}}}{{\mu }_{\text{L}}(1-\varepsilon )}\right)}^{\!-1\text{.}86}\left(\frac{{{d}_{\text{p}}\rho }_{\text{L}}\sigma }{{\mu }_{\text{L}}^{2}}\right)}^{\!2/3}$$

The capillary contribution to the pressure gradient is found to be dominant in the gas channeling regime but comparable to the viscous contribution in the large bubble regime. Combining the low interaction region data, a single correlation may be used to predict the two-phase friction well for this low interaction region (Eq. 16):

16 $${f}_{\text{TP}}-{f}_{\text{SP}}=0\text{.}19\,{({\text{Re}}_{\text{GS}}^{* })}^{0\text{.}68}{({\text{Re}}_{\text{LS}}^{* })}^{-1\text{.}85}{\text{Su}}_{\text{L}}^{2/3}$$

However, as it is observed the modified gas Reynolds number exponent in this correlation is much closer to its exponent at the gas channeling regime (Eq. 14) due to a higher pressure gradient or friction factor at high gas flow rates. Therefore, extracting separate correlations for each flow regime can predict the pressure gradient to gas superficial velocity dependency more accurately. We also obtained two separate correlations for the high interaction regime, where dispersed bubble (Eqs. 17–18) and pulse regimes (Eqs. 19–20) are identified:

17 $${f}_{\text{TP}}-{f}_{\text{SP}}=0\text{.}07\,{({\text{Re}}_{\text{GS}}^{* })}^{0\text{.}26}{({\text{Re}}_{\text{LS}}^{* })}^{-0\text{.}5}{\text{Su}}_{\text{L}}^{2/3}$$

18 $${\left(\frac{-\Delta P}{Z}\right)}_{\text{capillary}}=0\text{.}07\frac{\left(1-\varepsilon \right)}{{\varepsilon }^{3}}\left(\frac{{\rho }_{\text{L}}{U}_{\text{LS}}^{2}}{{d}_{\text{p}}}\right){\left(\frac{{\rho }_{\text{G}}{U}_{\text{GS}}{d}_{\text{p}}}{{\mu }_{\text{G}}(1-\varepsilon )}\right)}^{\!0\text{.}26}{{\left(\frac{{\rho }_{\text{L}}{U}_{\text{LS}}{d}_{\text{p}}}{{\mu }_{\text{L}}(1-\varepsilon )}\right)}^{\!\text{-}0\text{.}5}\left(\frac{{{d}_{\text{p}}\rho }_{\text{L}}\sigma }{{\mu }_{\text{L}}^{2}}\right)}^{\!2/3}$$

19 $${f}_{\text{TP}}-{f}_{\text{SP}}=0\text{.}11\,{({\text{Re}}_{\text{GS}}^{* })}^{0\text{.}35}{({\text{Re}}_{\text{LS}}^{* })}^{-0\text{.}82}{\text{Su}}_{\text{L}}^{2/3}$$

20 $${\left(\frac{-\Delta P}{Z}\right)}_{\text{capillary}}=0\text{.}11\frac{\left(1-\varepsilon \right)}{{\varepsilon }^{3}}\left(\frac{{\rho }_{\text{L}}{U}_{\text{LS}}^{2}}{{d}_{\text{p}}}\right){\left(\frac{{\rho }_{\text{G}}{U}_{\text{GS}}{d}_{\text{p}}}{{\mu }_{\text{G}}(1-\varepsilon )}\right)}^{0\text{.}35}{{\left(\frac{{\rho }_{\text{L}}{U}_{\text{LS}}{d}_{\text{p}}}{{\mu }_{\text{L}}(1-\varepsilon )}\right)}^{-0\text{.}82}\left(\frac{{{d}_{\text{p}}\rho }_{\text{L}}\sigma }{{\mu }_{\text{L}}^{2}}\right)}^{2/3}$$

Combining these two sets of data leads to Eq. 21 for predicting the two-phase friction factor in the high interaction region:

21 $${f}_{\text{TP}}-{f}_{\text{SP}}=0\text{.}12\,{({\text{Re}}_{\text{GS}}^{* })}^{0\text{.}32}{({\text{Re}}_{\text{LS}}^{* })}^{-0\text{.}81}{\text{Su}}_{\text{L}}^{2/3}$$

Here again the modified gas Reynolds number exponent is much closer to its exponent at the pulse regime (Eq. 19) where the two-phase friction factor is higher in comparison with the dispersed bubble regime. The experimental data scattering versus the fitted model data is higher in Eqs. 16 and 21 in comparison with Eqs. 12 and 14, and Eqs. 17 and 19 where separate correlations are provided for each single flow regime. The parity plots showing the accuracy of fitted correlations are presented in Supplementary Fig. 3.

We also analyzed the aircraft data^7^ which were mainly in the pulse flow and inertia-dominated regime, however, because it is not clear if the flow is fully developed in a few seconds of zero gravity duration, we did not add those data to our nonlinear regression model.

Figure 4 shows pressure gradients versus liquid superficial velocity at various gas superficial velocities. The slope change occurs at *U*_LS_ around 1 mm s^−1^ (Re*_LS_ = 3.6) which defines the approximate boundary between the low interaction (EB and GC regimes) and high interaction regions (DB and P regimes). This figure also shows there is a linear relationship between the pressure gradient and liquid superficial velocity in the high interaction region. Xu et al.^29^ also reported a similar behavior for their investigated small-packed bed reactor. A similar plot versus liquid-modified Reynolds number at various gas-modified Reynolds numbers is provided in Supplementary Fig. 4. The corresponding gas and liquid-modified Reynolds numbers for each operating gas and liquid superficial velocities are presented in Supplementary Tables 2 and 3. The normal gravity upflow data reported by Murugesan and Sivakumar^21^ are also plotted in Fig. 4. A slope change is observed in their pressure gradient data versus the liquid flow rate in each tested fixed gas flow rate. They found out the slopes and intercepts of the graphs vary with the flow regimes; however, they determined the flow regime boundaries using visual observation and expressed the boundaries in terms of two dimensionless numbers including all variables affecting the hydrodynamics of the two-phase cocurrent upflow. In Fig. 5a the capillary friction factor plots also show a slope change at Re*_LS_ ≈ 3.6–4 at three selected modified gas Reynolds numbers. These slope changes correspond to a transition from the gas channeling to the pulse flow regime.

The pressure gradient versus gas superficial velocity is plotted in Fig. 5b. The pressure gradient increases with increasing liquid superficial velocities all over the gas flow ranges. Two different slopes are observed in this figure with a transition point changing for each fixed superficial liquid velocity. The transition points correspond to the dispersed bubble to pulse flow regime boundary which is shown as an inclined line in the flow map presented in Fig. 3a. This transition occurs at a constant ratio of *U*_GS_ /*U*_LS_ which is similar to the results of Motil et al.^7^ who reported for each Suratman number, there exists a particular value for the ratio of Re_GS_/Re_LS_ where the bubble to pulse transition occurs. Our experimental data at very low liquid flow rates were not accurate enough and the slope change corresponding to the elongated bubble to gas channeling regime transition is plotted approximately in Fig. 3a.

**Fig. 7:**
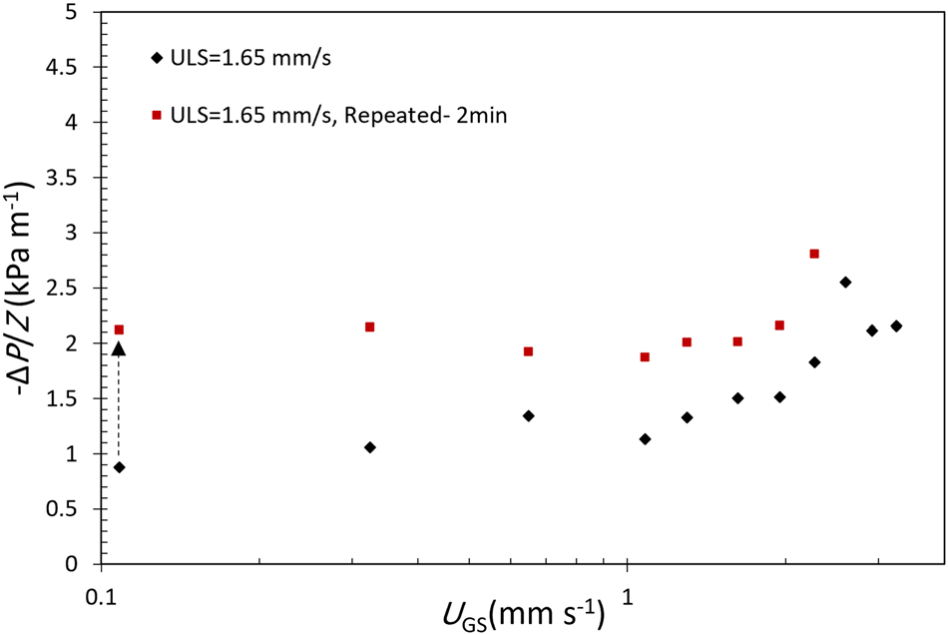
Comparison of measured pressure gradient at 30 s and 120 s into the experiment at a fixed superficial liquid velocity.

Some of the runs were repeated for a longer duration and data were collected after 120 s. Comparing these results with the shorter 30 s experiments show higher pressure gradients for the longer duration tests which verifies gas accumulation during flowing gas and liquid through the column even after reaching the defined steady-state condition (Fig. 7). Considering the length of the packed column (560 mm), the time required for traveling the column length for the liquid velocities of 1.03–1.65 mm s^−1^ is between 5.7 and 9 min which is much longer than 2 min reporting data collecting time. Therefore, the experiments need more time to reach a steady-state condition, especially at the low flow rates, and so the gas accumulation and pressure gradients would probably be higher than the shown values in Fig. 7 in case of giving a longer time for data collecting.

### Liquid and gas pre-flush tests

**Fig. 8:**
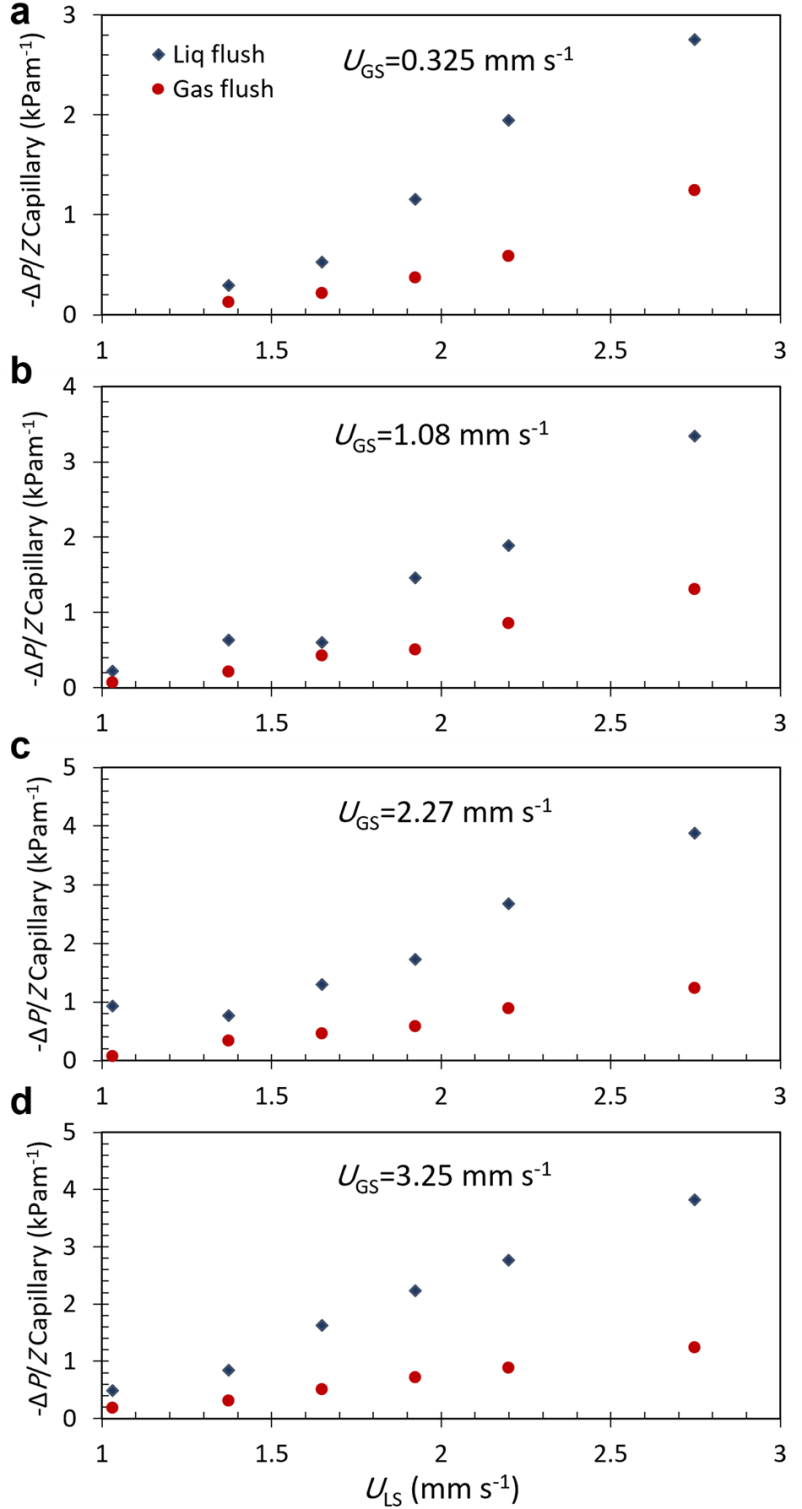
Capillary contribution to pressure gradient for both liquid and gas pre-flush as a function of liquid superficial velocity for four superficial gas velocities in the V–C regime. **a** U_GS_= 0.325 mm s^−1^; **b** U_GS_= 1.08 mm s^−1^; **c** U_GS_= 2.27 mm s^−1^; **d** U_GS_= 3.25 mm s^−1^.

Pre-flows of liquid flush, as well as gas flush, were used to establish similar initial conditions before testing. Figure 8 compares capillary pressure gradient vs. liquid superficial velocity at different gas superficial velocities for liquid flush tests with the gas flush ones inside the V–C regime. The higher pressure gradient in the liquid flush cases is attributed to having more trapped gas bubbles and therefore higher gas holdups in liquid flush tests. The removal of the stagnant bubbles by the gas flush preceding the test leads to lower gas hold-up and pressure gradients. The same result was also observed outside of the V–C regime and described in detail in Taghavi et al.^9^. However, the difference between liquid flush with gas flush capillary pressure data is more intensive at higher liquid superficial velocities inside the V–C regime.

### Hysteresis effects and gas hold-up in microgravity

**Fig. 9:**
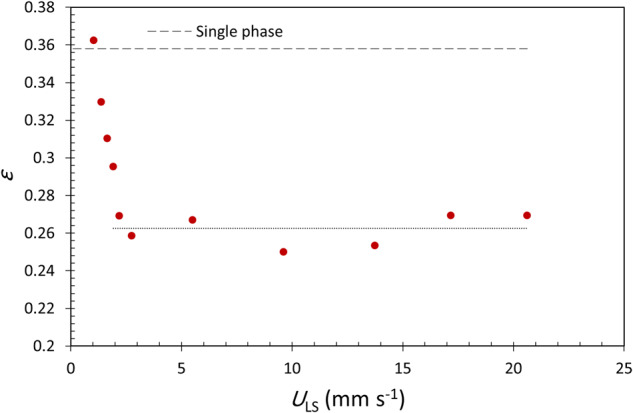
Estimated apparent porosity resulting from trapped bubbles in liquid pre-flush experiments inside and outside of the V–C regime.

The apparent bed porosity was estimated for different liquid flow rates by solving the single-phase Ergun equation for the porosity using the extrapolated pressure gradient at zero gas flow rate. As can be seen in Fig. 9 in the V–C regime (*U*_LS_ < 2 mm s^−1^), a sharp reduction in apparent porosity from 0.358 for the single-phase experiment to about 0.263 occurs due to trapped bubbles accumulation in the column. Beyond the V–C regime (*U*_LS_ > 2 mm s^−1^), the column reaches a semi-steady condition and the reduction in the apparent porosity is almost independent of the liquid flow rate and remains around 0.263 as reported by Taghvai et al.^9^. This apparent porosity corresponds to an average gas hold-up of 26.6% which is calculated as the ratio of trapped air volume to the total volume of bed void space. Taghavi and Balakotaiah^20^ also reported a gas holdup of 22% for their cocurrent upflow experiments in a 2 mm glass bead bed and in the limit of low gas flow rate. Saroha and Khera^30^ studied the hydrodynamics of fixed beds with cocurrent upflow and downflow in normal gravity and showed that variation of the total liquid holdup with liquid velocity in the range of 7–20 mm s^−1^ for a low gas velocity of 2 mm s^−1^ was negligible. In their experiments with 4 mm glass beads, the average liquid holdup for the upflow case was 26% (of bed volume) which corresponds to a gas holdup of 33% with respect to the bed void volume of 0.39; Similarly, for the downflow case, the average liquid holdup was 23% which corresponds to a gas holdup of 41%.

**Fig. 10:**
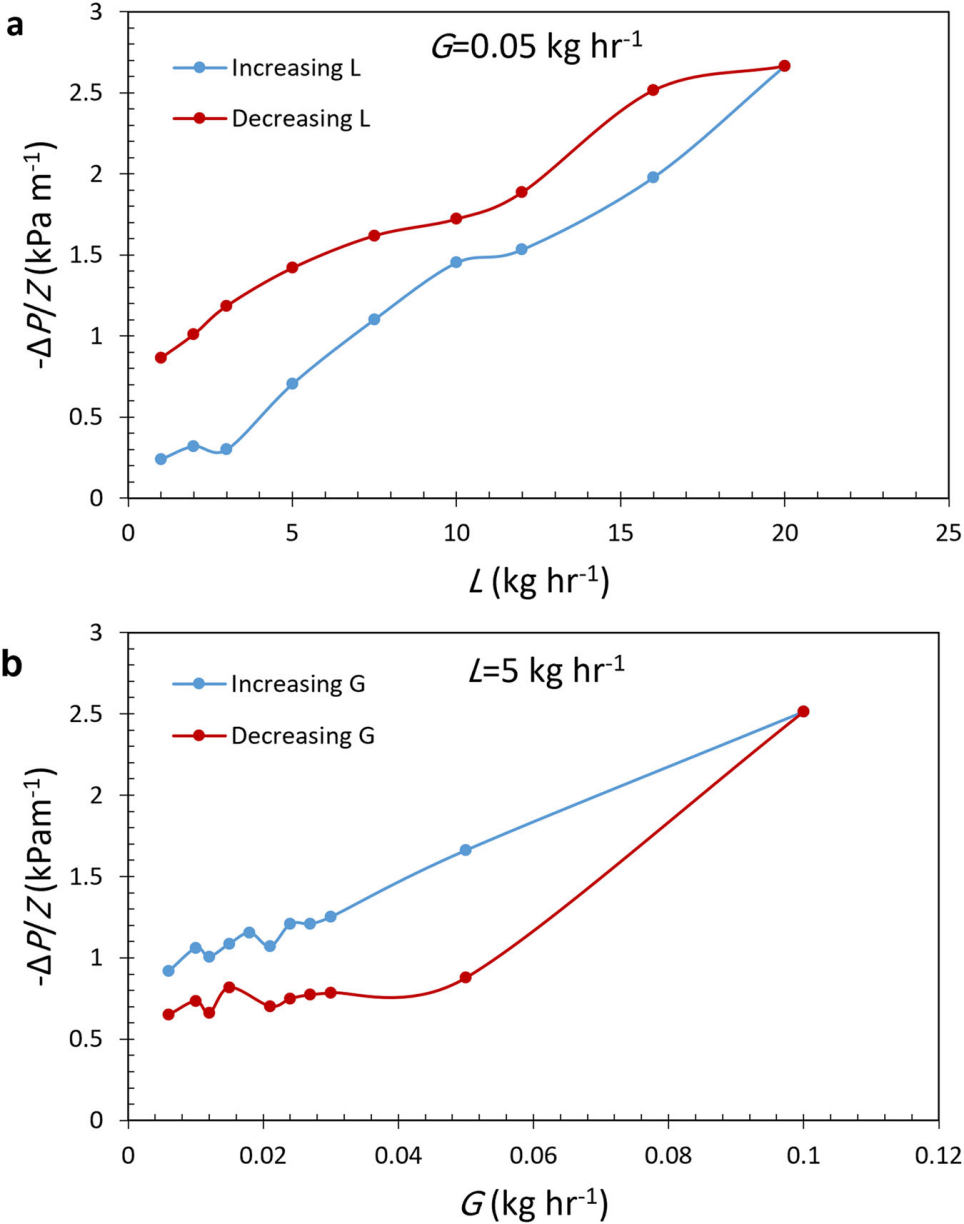
Measured pressure gradient in hysteresis experiments. **a** Constant gas flow rate; **b** Constant liquid flow rate.

Increasing and then decreasing the liquid (gas) flow rate, at a fixed gas (liquid) flow leads to different values for the liquid holdup and pressure gradient. Figure 10 shows the existence of the hysteresis effect within the V–C regime for both constant gas flow rate (Fig. 10a) and constant liquid flow rate (Fig. 10b) experiments.

However, in our first series of PBRE experiments^8^ which were performed outside of the V–C regime, the hysteresis effect (relative magnitude) in the measured pressure gradient was found to be negligible over the range of studied flow conditions. A comparison between the transient flow pressure gradients with the steady-state flow pressure gradients is also presented in Supplementary Fig. 5.

## Discussion

In this article, we have presented a comprehensive analysis of the available flow pattern and pressure drop data for gas–liquid two-phase flow through packed beds in microgravity. The data analyzed included aircraft experiments as well as two sets of experiments aboard the International Space Station. The major results of our analysis may be summarized as follows: (i) For gas and liquid flow rates of interest in most microgravity applications, there are four major flow patterns, namely, dispersed bubble, pulse, elongated/large bubble regime, and gas continuous regime. (ii) We have developed an accurate flow pattern map for the nitrogen-water system based on the change in slope of the pressure gradient with either gas or liquid flow rate. (iii) We have shown that the dependence of the pressure gradient (or friction factor) on gas and liquid flow rates (or Reynolds numbers) is different in each flow regime. Hence, a separate correlation is required to make accurate predictions of the pressure drop in each flow regime, and such correlations are presented here for the first time. (iv) As can be expected, it is found that capillary effects dominate the overall pressure drop, especially at lower flow rates. The capillary contribution dependence on gas and liquid flow rates is found to be different in different flow regimes. (v) Our analysis indicates that the gas hold-up is a function of bed history, especially at low gas and liquid flow rates. Hysteresis effects are observed at low gas and liquid flow rates but the relative magnitude of the hysteresis becomes negligible at higher flow rates. (vi) Our data at very low liquid and gas flow rates also indicated that the large bubble regime is intermittent in nature due to the slow accumulation of gas in the bed with times scales of the order of several minutes (depending on the flow rates) and is dominated by capillary effects.

As stated in the introduction, the accuracy of our pressure measurements or the duration of any experiment at a fixed gas and liquid flow rate was not sufficient to characterize the large bubble regime in any detail quantitatively. However, the limited data indicate that this flow regime is intermittent with a frequency of about 0.01 Hz or smaller. [This approximate frequency was determined from observation of the pressure traces of the few long-duration experiments]. In contrast, the pulse frequency in both the aircraft and ISS experiments was of the order of 4 Hz, see Salgi et al.^31^.

For future studies of gas-liquid flow in packed beds at very low gas and liquid flow rates (or Reynolds numbers much smaller than unity) in both normal and microgravity, the authors recommend the use of longer beds with more accurate pressure gradient measurement. In addition, longer duration experimental measurements of the order of several minutes to an hour (depending on the flow rates) are recommended in order to assess and capture the intermittent phenomenon associated with gas accumulation. Finally, it should be pointed out that due to experimental limitations on ISS, we were not able to measure the gas void fraction in the bed. For future experiments either in normal or microgravity, the authors strongly recommend that the pressure gradient and flow visualization be supplemented with an accurate void fraction sensor for a better assessment of gas accumulation and its impact on flow patterns and pressure gradient.

### Reporting summary

Further information on research design is available in the Nature Research Reporting Summary linked to this article.

## Supplementary information

SUPPLEMENTAL MATERIAL

Reporting Summary

## Data Availability

The datasets generated and/or analyzed during the current study are available (to registered users) at the NASA Physical Sciences Informatics (PSI) website: https://www.nasa.gov/PSI.
