# Supplementary material for: The international space station packed bed reactor experiment: capillary effects in gas-liquid two-phase flows
Source: NPJ Microgravity. 2023 Jul 18;9:55. doi: 10.1038/s41526-023-00302-2 (PMC10354015; doi:10.1038/s41526-023-00302-2)
Supplement: Supplementary file 1 — SUPPLEMENTAL MATERIAL [file 41526_2023_302_MOESM1_ESM.pdf]

SUPPLEMENTARY DISCUSSION

This Supplementary Material aims to present additional experimental data and analysis of PBRE ISS experiments.

Pressure Gradient Measurements and Flow Regimes

Supplementary Figure 1 shows an approximate map of the flow regimes for the water-N2 system observed in the microgravity experiment with 2 mm packing size versus the gas and liquid modified Reynolds numbers.

Supplementary Figure 2 compares the microgravity flow regime map with the normal gravity downflow (Tosun) and normal gravity upflow (Murugesan and Sivakumar) maps. A good overlap is observed between all three data series in the pulse and dispersed bubble flow regimes at high liquid flow rates.

Parity plots for the gas channeling and pulse flow regimes are presented in Supplementary Figures 3 (a) and (b), respectively. These plots show that the flow regime correlations fit the experimental data well. Supplementary Figure 3 (c) demonstrates how well the models for two adjoining gas channeling and Pulse regimes can predict pressure drop on the regime transition borderline.

The measured pressure gradient versus liquid Reynolds number is provided in Supplementary Figure 4 which shows the low to high interaction regime transition boundary at Re^*_{LS}=3.6.

Supplementary Figure 5 compares the transient flow pressure gradients obtained with increasing and decreasing the gas flow rate with the steady-state flow pressure gradients obtained following a liquid flush and gas flush pre-flows. Transient flow pressure gradients are higher than the steady-state ones due to a higher gas accumulation throughout the bed during these experiments.

Supplementary Table 1 summarizes and compares the operational parameters and fluid properties used in the PBRE and PBRE-2 tests.

The gas and liquid superficial velocities in terms of modified Reynolds numbers, flow rates, and fluxes are presented in Supplementary Tables 2 and 3, respectively.

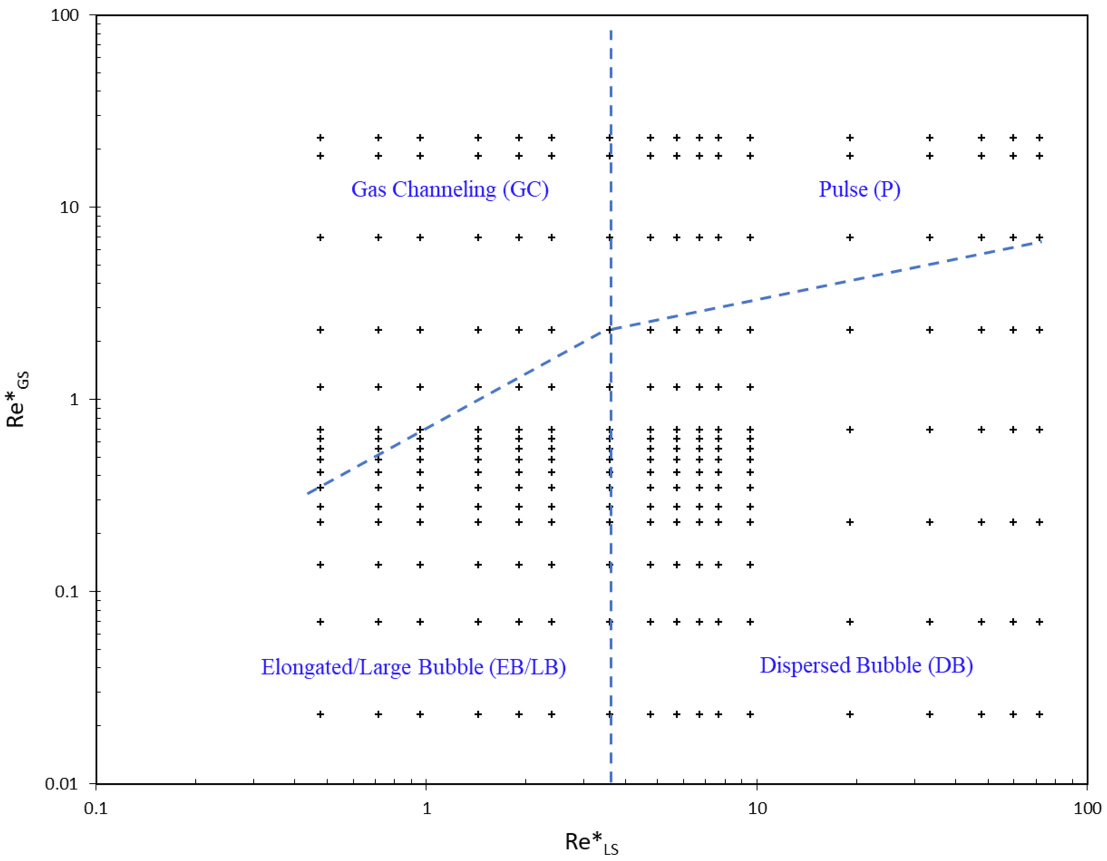

Supplementary Figure 1. Approximate map of the flow regimes for the water-N_2 system observed in the microgravity PBRE experiment versus modified Reynolds numbers.

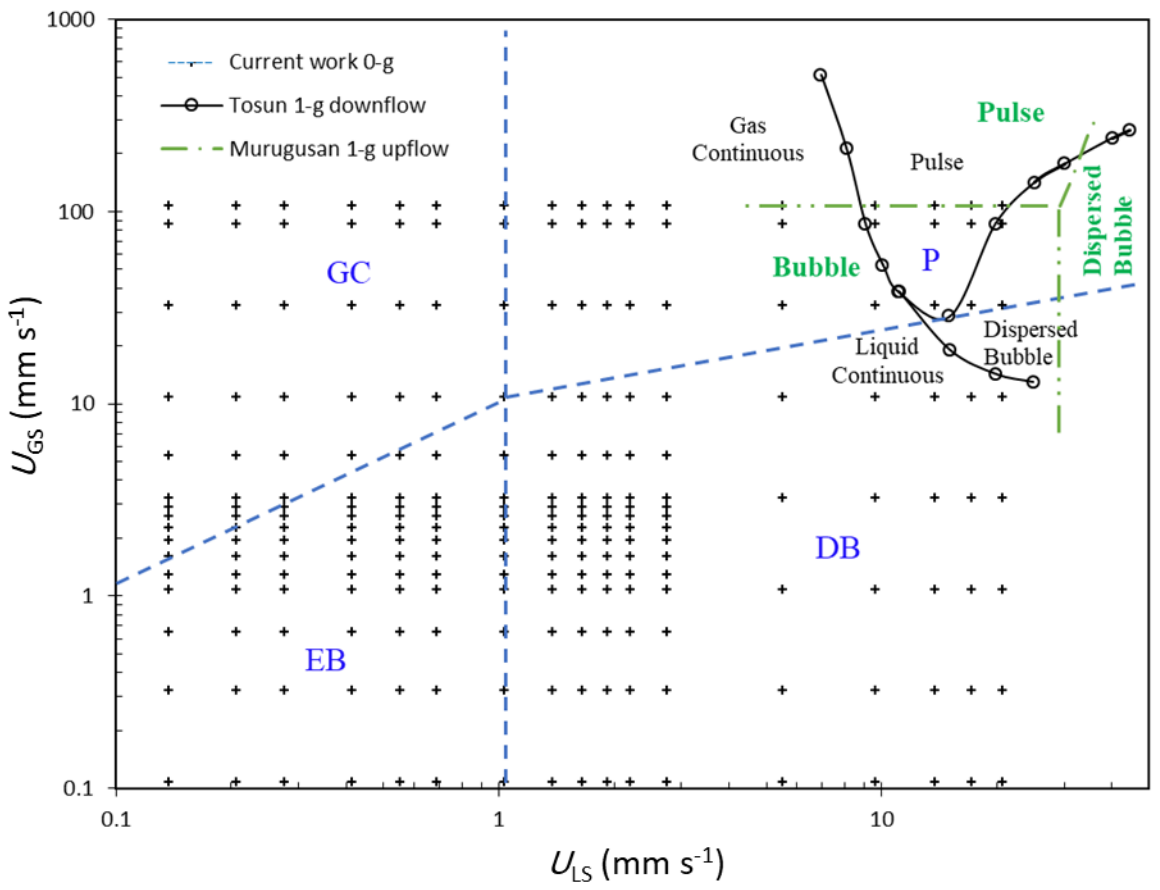

Supplementary Figure 2. Comparison of the microgravity flow map (blue dashed lines) with the normal gravity downflow (black solid lines) and upflow maps (green dashed lines) versus superficial gas and liquid velocities.

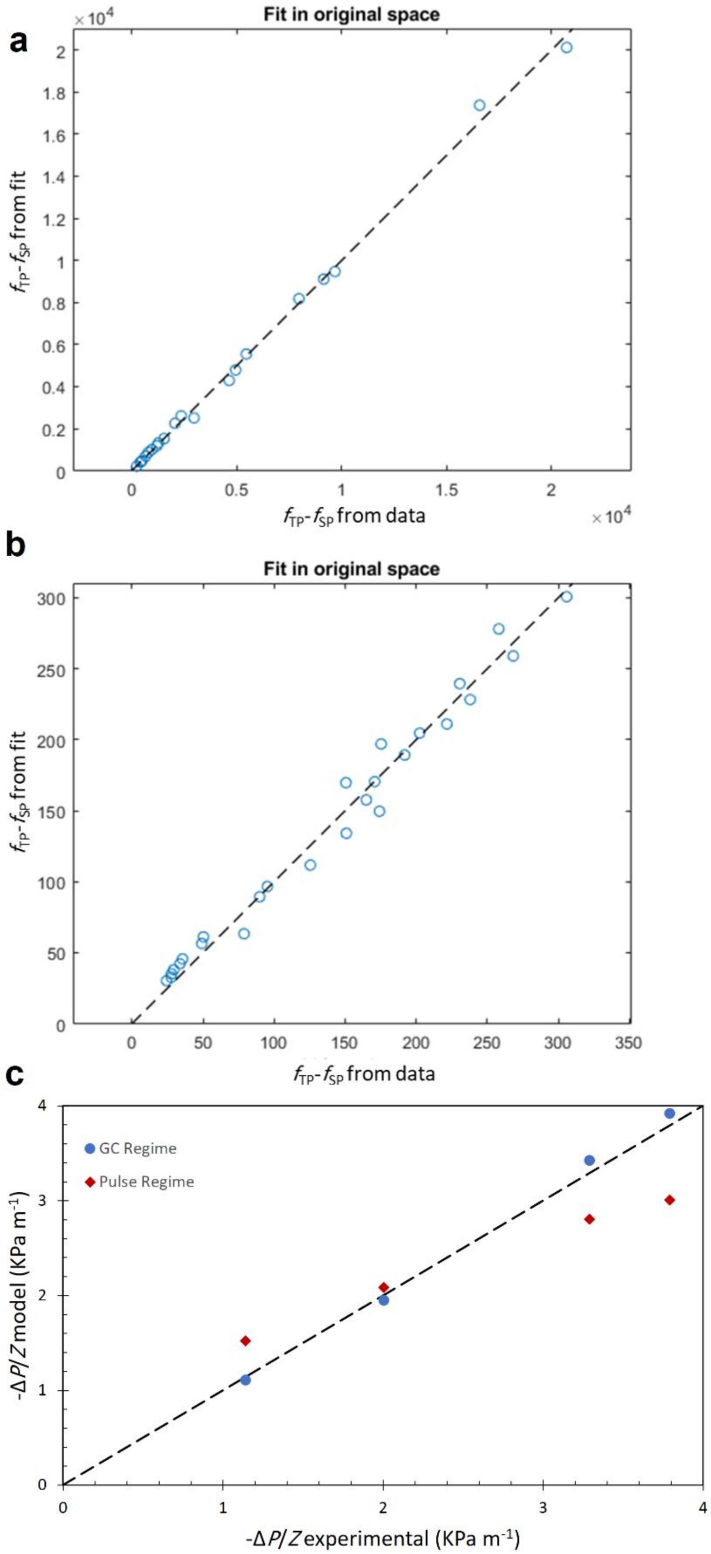

Supplementary Figure 3. Parity plot for the **(a)** gas channeling regime; **(b)** pulse regime; **(c)** gas channeling and pulse regimes for the borderline data.

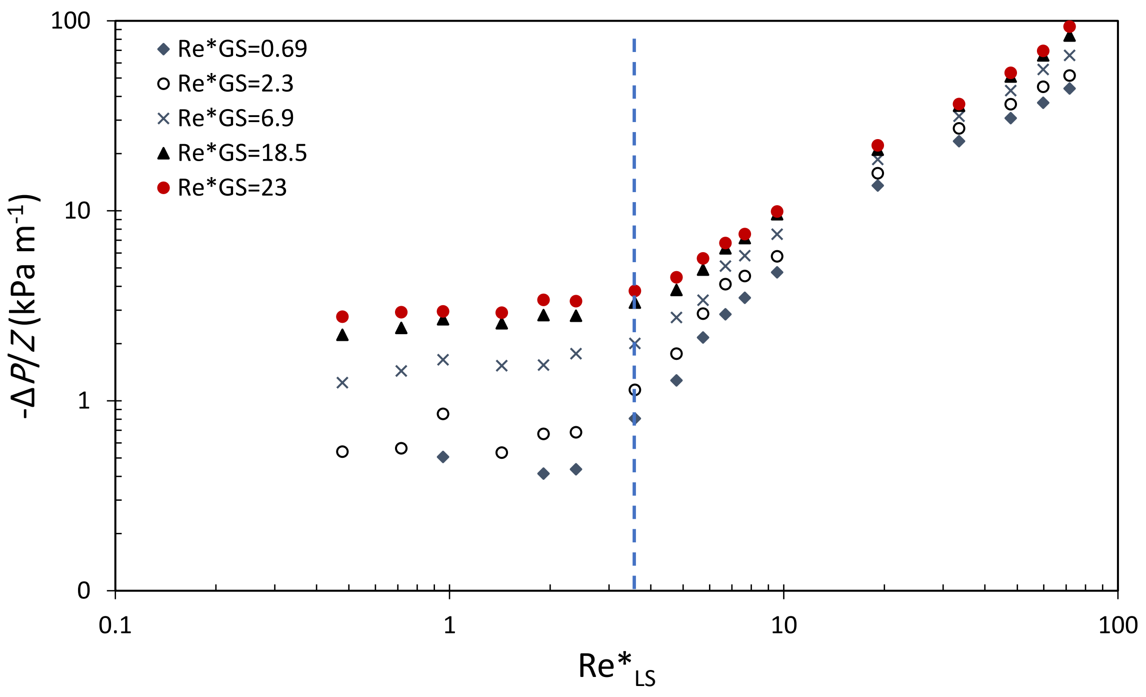

Supplementary Figure 4. Measured pressure gradient versus modified liquid Reynolds number.

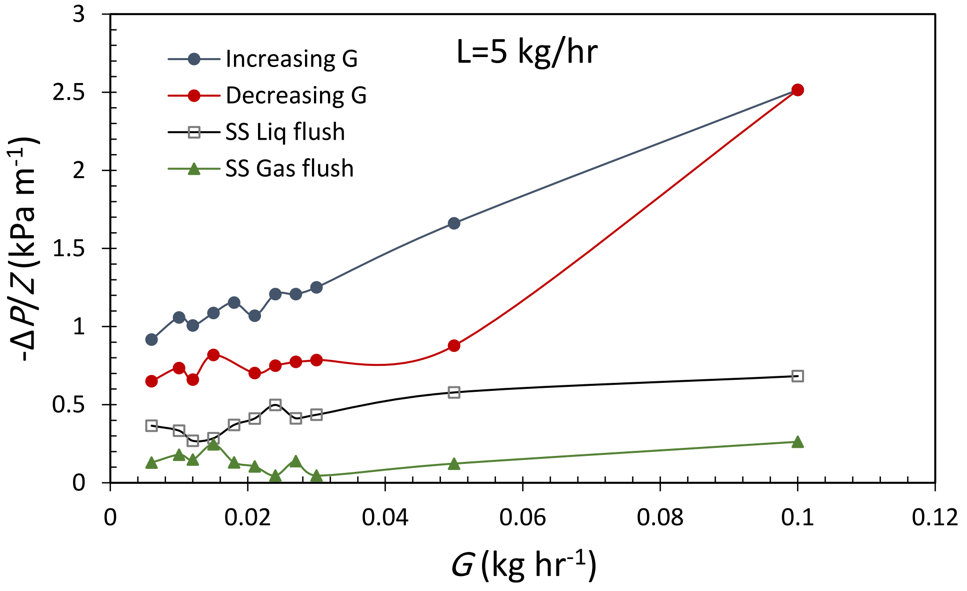

Supplementary Figure 5. Comparison of the transient flow experiments with the steady-state flow liquid flush and gas flush experiments versus gas flow rate at a fixed liquid flow rate.

Supplementary Table 1. PBRE versus PBRE-2 parameters

Parameter	PBRE Tests	PBRE-2 Tests
Re^*_{GS}	0.1 - 33	0.02 - 23
Re^*_{LS}	1.5 – 105	0.5 – 72
G	$4x10^{-4}$ – 0.14 kg $m^{-2}s^{-1}$	$1.4x10^{-4}$ – 0.14 kg $m^{-2}s^{-1}$
L	0.25 – 20 kg $m^{-2}s^{-1}$	0.14 – 20 kg $m^{-2}s^{-1}$
g	10^{-4} m s^{-2}	10^{-4} m s^{-2}
**ε** (porosity)	0.34	0.358
μ_G	$1.85x10^{-5}$ kg $m^{-1}s^{-1}$	$1.85x10^{-5}$ kg $m^{-1}s^{-1}$
μ_L	$8.9x10^{-4}$ kg $m^{-1}s^{-1}$	$8.9x10^{-4}$ kg $m^{-1}s^{-1}$
σ	0.065 – 0.070 kg s^{-2}	0.070 kg s^{-2}
d_P (particle diameter)	3 mm	2 mm
Packing material	Glass and Teflon	Glass
Typical test duration	30 s	30 and 120 s
Pre-flow	Liquid	Liquid and gas

Supplementary Table 2. Gas superficial velocity in terms of modified Reynolds number, flow rate, and mass flux

U_{GS} (mm s^{-1})	Re^*_{GS}	Gas flow rate (kg hr^{-1})	Gas mass flux (kg $m^{-2}s^{-1}$)
0.108	0.023	0.001	1.37E-04
0.325	0.069	0.003	4.11E-04
0.649	0.138	0.006	8.22E-04
1.082	0.231	0.01	1.37E-03
1.298	0.277	0.012	1.64E-03
1.623	0.346	0.015	2.05E-03
1.947	0.415	0.018	2.47E-03
2.272	0.484	0.021	2.88E-03
2.596	0.554	0.024	3.29E-03
2.921	0.623	0.027	3.70E-03
3.246	0.692	0.03	4.11E-03
5.409	1.153	0.05	6.85E-03
10.82	2.307	0.1	1.37E-02
32.46	6.921	0.3	4.11E-02
86.55	18.46	0.8	1.10E-01
108.19	23.07	1	1.37E-01

Supplementary Table 3. Liquid superficial velocity in terms of modified Reynolds number, flow rate, and mass flux

U_{LS} (mm s^{-1})	Re^{*}_{LS}	Liquid flow rate (kg hr^{-1})	Liquid mass flux (kg m^{-2}s^{-1})
0.137	0.478	1	0.137
0.206	0.716	1.5	0.205
0.275	0.955	2	0.274
0.412	1.433	3	0.411
0.550	1.911	4	0.548
0.687	2.388	5	0.685
1.031	3.582	7.5	1.027
1.374	4.776	10	1.370
1.649	5.732	12	1.644
1.924	6.687	14	1.918
2.199	7.642	16	2.192
2.748	9.553	20	2.740
5.496	19.11	40	5.480
9.619	33.44	70	9.590
13.74	47.76	100	13.70
17.18	59.71	125	17.12
20.61	71.65	150	20.55
